# Alpha-Naphthoflavone as a Novel Scaffold for the Design of Potential Inhibitors of the APH(3’)-IIIa Nucleotide-Binding Site of *Enterococcus faecalis*

**DOI:** 10.3390/microorganisms11092351

**Published:** 2023-09-20

**Authors:** Juliana Carolina Amorim, Juan Marcelo Carpio

**Affiliations:** Unidad Académica de Salud y Bienestar, Universidad Católica de Cuenca, Av. Las Américas, Cuenca 010105, Ecuador; julianacarolinaamorim@gmail.com

**Keywords:** *Enterococcus faecalis*, antibiotic resistance, aminoglycosides, aminoglycoside-modifying enzymes, APH(3’)-IIIa, flavones, naphthoflavones

## Abstract

The spread of nosocomial infections caused by antibiotic-resistant *Enterococcus faecalis* is one of the major threats to global health at present. While aminoglycosides are often used to combat these infections, their effectiveness is reduced by various resistance mechanisms, including aminoglycoside modifying enzymes, and there are currently no drugs to inhibit these enzymes. To address this issue, this study was conducted to identify potential aminoglycoside adjuvants from a database of 462 flavones. The affinity of these molecules with the nucleotide-binding site (NBS) of aminoglycoside phosphotransferase type IIIa of *E. faecalis* (EfAPH(3’)-IIIa) was evaluated, and the five molecules with the highest binding energies were identified. Of these, four were naphthoflavones, suggesting that their backbone could be useful in designing potential inhibitors. The highest-ranked naphthoflavone, 2-phenyl-4H-benzo[h]chromen-4-one, was modified to generate two new derivatives (ANF2OHC and ANF2OHCC) to interact with the NBS similarly to adenine in ATP. These derivatives showed higher binding free energies, better stability in molecular dynamics analysis and superior pharmacokinetic and toxicological profiles compared to the parent molecule. These findings suggest that these alpha-naphthoflavone derivatives are potential inhibitors of EfAPH(3’)-IIIa and that this core may be a promising scaffold for developing adjuvants that restore the sensitivity of aminoglycosides.

## 1. Introduction

According to the World Health Organization, antibiotic resistance remains one of the most significant global threats to public health [1]. This problem primarily stems from the inappropriate use of available antibiotics, which leads to the development of bacterial resistance mechanisms [1,2]. Enterococci, particularly *Enterococcus faecalis*, are a major concern in the hospital environment [3,4]. Although part of the human gastrointestinal microbiota, this species has become a constant threat, especially to hospitalized patients because of the risk of developing bacteremia, endocarditis, and urinary tract and intra-abdominal infections [4,5,6]. The limited susceptibility of enterococci to antibiotics is due to both intrinsic and extrinsic resistance mechanisms [7,8]. The intrinsic mechanisms are mainly linked to the widespread utilization of aminoglycosides, cephalosporins, clindamycin, sulfamethoxazole, and trimethoprim antibiotics [8]. Conversely, the extrinsic resistance mechanisms arise from the acquisition of vancomycin, ampicillin, and aminoglycoside resistance genes or mutations through horizontal transfer in enterococci [8,9]. Although aminoglycosides were previously the drugs of first choice for certain infections caused by enterococci, their progressive loss of efficacy has resulted in their replacement by other antibiotics [7,9,10].

Currently, there are several known mechanisms of bacterial resistance to aminoglycosides. Among the main are: (1) reduced permeability of the bacterial outer membrane, or ineffective inner membrane transport leading to decreased intracellular concentration of aminoglycosides; (2) efflux of aminoglycosides from the cell through pumps; (3) structural mutations in ribosomes; (4) activity of methyltransferases that interfere with the binding of aminoglycosides to 16S rRNA; and (5) expression of aminoglycoside-modifying enzymes (AMEs) [7,9,10,11]. The drug inactivation by AMEs is the mechanism that has received the most attention due to the frequency of resistance conferred to enterococci [9]. These enzymes inactivate aminoglycosides by transferring a functional group to them. AMEs are divided into three families based on the chemical nature of the group they transfer: (1) aminoglycoside O-nucleotidyltransferases (ANTs) transfer a nucleoside monophosphate to a hydroxyl group of the antibiotic; (2) aminoglycoside N-acetyltransferases (AACs) transfer an acetyl group to the amine group of the aminoglycoside; and (3) aminoglycoside O-phosphotransferases (APHs) transfer a gamma phosphate to a hydroxyl group of the antibiotic. APHs are the most widespread AME among pathogens [8,9,10]. APH(3’)-IIIa (hereinafter referred to as EfAPH3’-IIIa) is one member of the latter family that has been extensively studied in *E. faecalis* due to its wide variety of substrates.

As bacterial resistance to antibiotics becomes more prevalent and the search for new antibiotics declines, developing adjuvants that can restore the efficacy of traditional antibiotics is a promising alternative [12,13]. Phytochemical-based drug discovery, which has yielded significant results, remains an important trend [14,15,16,17,18,19,20]. Among the natural products with verified effects on various bacterial targets, flavonoids are a prominent group [21,22]. These compounds have a core structure of C6-C3-C6 (A, C, and B rings, respectively) with an oxygen atom at position 1 and a ketone functional group at position 4 of the C ring [23,24,25]. Flavonoids are classified into flavonols, flavanones, flavanonols, and flavones based on their structural characteristics [26]. Several natural flavonoids are decorated with functional groups such as hydroxyl, methyl, and methoxyl [27]. Some flavonoids act as competitive inhibitors of enzymes that, like APH3’-IIIa, transfer phosphate groups from ATP, including some human kinases [28,29]. In addition, some flavones have shown synergistic effects with antibiotics on bacteria such as *Pseudomonas aeruginosa*, and without exerting antibacterial activity, a property that makes them suitable for the development of antibiotic adjuvants [30,31]. To date, there are no reports on the effect of flavones applied as adjuvants to aminoglycosides on *E. faecalis*. In this study, we selected 462 flavones from the ChemSpace database to identify potential inhibitors that act at the nucleotide-binding site (NBS) of APH3’-IIIa from *E. faecalis.*

## 2. Results and Discussion

### 2.1. Virtual Screening Analysis of Flavones in the Nucleotide-Binding Site of EfAPH3’-IIIa

To validate the parameters used in the docking-based virtual screening protocol, the co-crystallized ligand (CKI-7) was re-docked using AutoDock Vina-1.1.2. Figure S1A presents the results of this analysis, which shows an RMSD value of 0.28 Å, indicating that the software can reproduce the experimental pose of the co-crystallized ligand. 

The virtual screening was performed on a total of 462 flavones, including 42 flavone glycosides, five naphthoflavones and the remaining were flavones modified with substituents of different chemical natures (Supplementary Table S1). Notably, four of the five best-scoring (BS) molecules are naphthoflavones. Moreover, unlike other molecules in the dataset, these naphthoflavones do not possess polar substituents. 

The top-scoring molecule was CSSB00000067355 (alpha-naphthoflavone, BS-1), followed by CSSB00000067363 (beta-naphthoflavone, BS-2), CSSB00160825754 (a flavone, BS-3), CSSB00011876001 (a beta-naphthoflavone, BS-4), and CSSB00102592853 (a beta-naphthoflavone, BS-5). Importantly, all of these molecules demonstrated higher binding energy compared to the co-crystallized ligand CKI-7, as indicated in Table 1.

**Figure 1:**
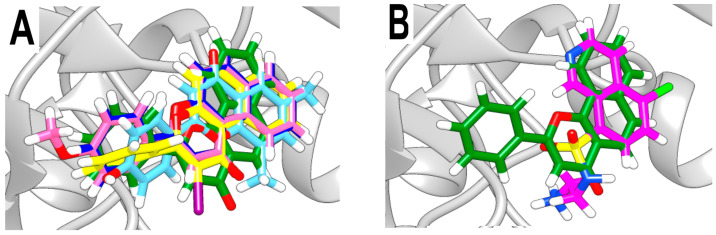
Overlap between the best-scored flavones and CKI-7. (**A**) Overlap among the five best-scored flavones. (**B**) Overlap between CKI-7 and CSSB00000067355 (alpha-naphthoflavone, BS-1). The colors magenta, green, dark blue, light blue, light pink and yellow represent, respectively, the ligand CKI-7 and the flavones CSSB00000067355, CSSB00000067363, CSSB00160825754, CSSB00011876001 and CSSB00102592853.

Furthermore, all BS flavones showed a similar orientation, with their phenyl group (ring B) projecting outward from the NBS of 3Q2J, as depicted in Figure 1A. However, in a comparative analysis of these five molecules, a greater overlap was found between the naphthalene ring of BS-1 (2-phenyl-4H-benzo[h]chromen-4-one, hereinafter referred to as ANF) and the isoquinoline ring of CKI-7, Figure 1B. Given this observation, and knowing that CKI-7 and the adenine moiety of ATP establish several similar interactions at the NBS of EfAPH3’-IIIa [12], ANF was chosen for subsequent analyses. 

### 2.2. Molecular Docking Analysis of 3Q2J Complexed with CKI-7 and ANF

**Figure 2:**
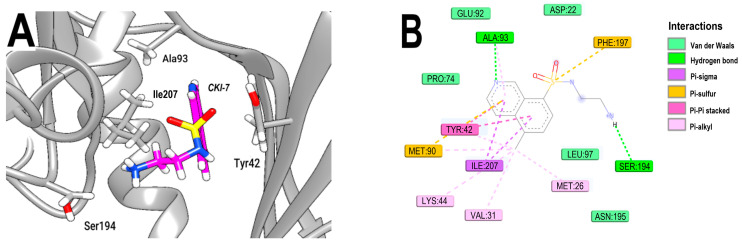
Analysis of the crystallographic pose of CKI-7 in the NBS of 3Q2J. (**A**) Three-dimensional interaction representation. (**B**) Two-dimensional interaction diagram.

Some of the main interactions of CKI-7 with NBS of 3Q2J include H-bonds involving the amino group of its aminoethyl chain (with SER194) and the nitrogen in the pyridine ring (with ALA93). On the other hand, its isoquinoline ring formed Pi–Pi stacking and Pi–sigma interactions with the aromatic ring of TYR42 and with ILE207, respectively (Figure 2A,B).

**Figure 3:**
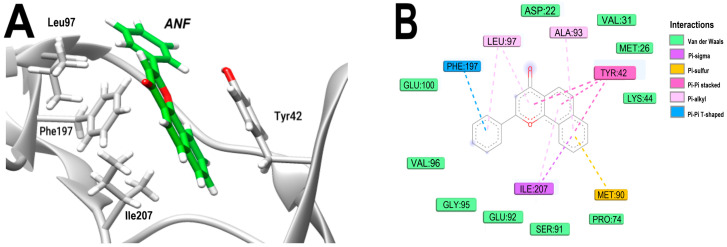
Analysis of the docked pose of the ANF in the NBS of 3Q2J. (**A**) Three-dimensional interaction representation. (**B**) Two-dimensional interaction diagram.

Two-dimensional representation of the 3Q2J-ANF complex reveals Pi–sigma interactions with ILE207 through its naphthalene ring and absence of hydrogen bonds. Additionally, due to its parallel orientation towards the aromatic ring of TYR42, ANF forms Pi–Pi stacking interactions. Furthermore, its B-ring establishes Pi–Pi T-shape interactions with PHE197, as illustrated in Figure 3A,B. Notably, both TYR42 and PHE197 are crucial residues for the interactions between 3Q2J and ANF, and they have been identified as functionally important for the enzyme activity [12,32]. Indeed, TYR42 is highly conserved in APH3’-IIIa and plays a critical role in catalysis and the formation of stacking interactions with the adenine moiety of nucleotides [12]. A notable difference between this enzyme and kinases is that in the latter TYR42 is replaced by ALA, which is a distinctive feature that can be exploited to develop more specific inhibitors directed against APH3’-IIIa [32].

### 2.3. Pharmacokinetic and Toxicological Analyses of ANF

The results presented in Table 2, based on SwissADME predictions, indicate high gastrointestinal absorption of ANF, making it potentially suitable for oral administration. However, its ability to cross the blood–brain barrier (BBB) could lead to adverse effects on the central nervous system [33]. In addition, ANF has the potential to inhibit CYP1A2 and CYP2C9, enzymes responsible for the metabolism of several drugs. Consequently, this molecule could generate interactions with substances metabolized by these cytochromes [34]. These predictions are consistent with previous studies that have shown that several flavonoids, including certain naphthoflavone derivatives, can modulate the activity, expression levels and stability of several cytochromes [35,36].

Additionally, a toxicity analysis conducted using the ProTox-II web server reveals that ANF is not hepatotoxic, immunotoxic, or mutagenic. However, it does exhibit carcinogenic and cytotoxic potential. Collectively, these results indicate that ANF possesses some desirable features for the development of EfAPH3’-IIIa inhibitors. However, it also possesses toxic potential and certain characteristics that may lead to adverse effects. 

### 2.4. Design and Molecular Docking Analyses of ANF Derivatives

**Figure 4:**
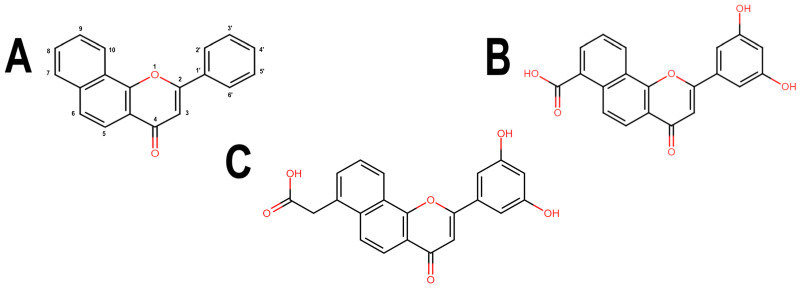
Chemical structures of BS-1 and its derivatives. (**A**) BS-1 named ANF. Atom numbering used in the current study is indicated. (**B**) Naphthoflavone derivative named ANF2OHC. (**C**) Naphthoflavone derivative named ANF2OCC.

To overcome the inadequate pharmacokinetic and toxicological profile of ANF (Figure 4A), in the next step, it was decided to modify its backbone while preserving its key interactions at the catalytic site. From a pharmacokinetic perspective, molecules such as ANF, which exhibit low polarity, lack electrical charges and possess low molecular weight, tend to have a higher propensity to cross the BBB [37,38], as indicated by predictions from SwissADME. Considering this, it was decided to introduce polar groups in ANF to modify these physicochemical properties. To determine the most suitable positions for substitution, an analysis of the NBS structure was carried out so that these groups additionally promote the formation of interactions. Figure S1B shows the H-bond acceptor and donor groups of the NBS residues close to ANF. 

Based on the aforementioned analyses, two hydroxyl groups were introduced at positions 3’ and 5’ of the phenyl ring of ANF, resembling the structure of a known flavone, 3’,5’-dihydroxyflavone (PubChem CID: 45933941). These modifications were aimed at increasing the polarity of ANF and establishing additional interactions with nearby H-bond acceptor groups (carboxyl groups of GLU92 and GLU100 and the carbonyl group of the peptide bond of GLY95) and a H-bond donor group (NH group of the peptide bond of LEU97). While it is visually evident that further hydroxyl substitutions at positions 3’ and 4’, as well as 3’, 4’, and 5’, could be possible, they were not tested to avoid the formation of a catechol moiety. 

To further decrease the likelihood of crossing the BBB [39], additional modifications of the derivatives were conducted. One of them carries an ionizable carboxyl directly at position 7 of the naphthalene ring and another derivative with the carboxyl attached to this same carbon via a methyl group to bring the carboxyl closer to the H-bond acceptor groups within the NBS (nucleotide-binding site). These ANF derivatives will be referred to as ANF2OHC (Figure 4B) and ANF2OHCC (Figure 4C), respectively. Importantly, the presence of carboxyl groups in drugs is not infrequent and more than 450 drugs, including antibiotics such as beta-lactams and fluoroquinolones, contain this group in their structures [40]. 

### 2.5. Blind Docking Analysis

To assess whether ANF2OHC and ANF2OHCC would show higher affinity for binding sites other than the NBS, including the aminoglycoside site, blind docking analysis was performed. The results reveal that both molecules are located within the same NBS of the enzyme, indicating selectivity for the nucleotide-binding site (NBS, Figure S2A,B).

### 2.6. Molecular Docking Analysis of 3Q2J Complexed with ANF2OHC and ANF2OHCC

The docking analysis of the best pose of ANF2OHC within the NBS reveals that the incorporation of the hydroxyl and carboxyl groups enhances the binding affinity of this ligand, resulting in a value of −11.0 kcal/mol. Furthermore, this modification only induces a slight shift in the orientation of the naphthoflavone backbone compared to that of ANF, Figure S3A. It is noteworthy that when the carboxyl group was introduced at positions 8, 9, or 10 of the naphthalene rings, the binding energies of these derivatives decreased to -9.6, −9.9, and −9.6 kcal/mol, respectively. As a result, further analysis of these derivatives was not performed. 

**Figure 5:**
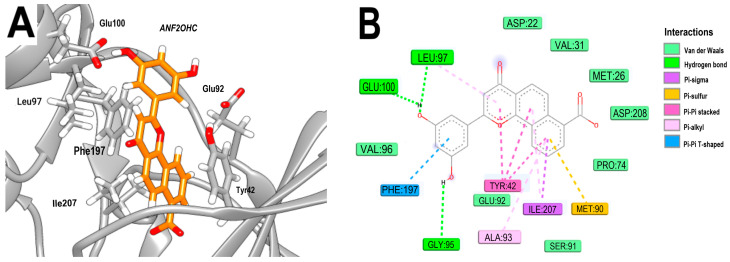
Analysis of the docked pose of the ANF2OHC in the NBS of 3Q2J. (**A**) Three-dimensional interaction representation. (**B**) Two-dimensional interaction diagram.

The analysis of the 3Q2J-ANF2OHC complex demonstrates that the hydroxyl groups establish hydrogen bonds with GLU92, LEU97, and GLU100. Moreover, interactions observed in the 3Q2J-ANF complex involving the naphthoflavone backbone, such as Pi–Pi stacking with TYR42 (involving the naphthalene ring) and Pi–Pi T-shaped interactions with PHE197 (involving the phenyl ring) are preserved in the 3Q2J-ANF2OHC complex. However, under the conditions tested, the incorporated carboxyl group does not form any interaction in the NBS (Figure 5A,B). 

**Figure 6:**
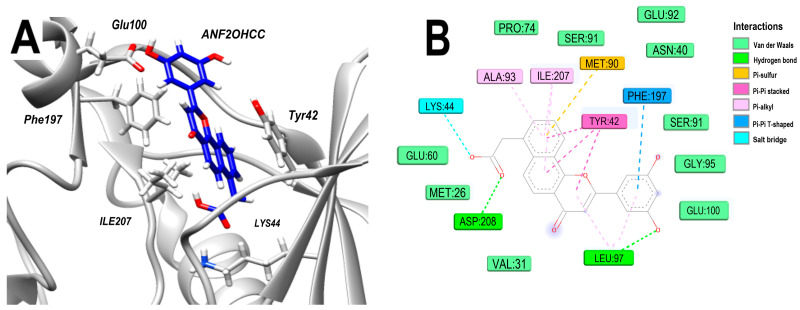
Analysis of the docked pose of the ANF2OHCC in the NBS of 3Q2J. (**A**) Three-dimensional interaction representation. (**B**) Two-dimensional interaction diagram.

The docking analysis of ANF2OHCC in complex with 3Q2J reveals a binding energy of −10.3 kcal/mol. This value is close to that of ANF and lower than ANF2OHC, but this ligand preserves multiple key interactions of the backbone because its pose remains virtually unchanged relative to that of ANF2OHC, Figure S3B. Interaction analysis shows that a hydroxyl group form a hydrogen bond with LEU97. Moreover, several interactions were observed in the 3Q2J-ANF and 3Q2J-ANF2OHC complexes, such as the Pi–Pi stacking with TYR42 (involving the naphthalene ring) and the Pi–Pi T-shaped interactions (between the phenyl ring and PHE197), are conserved in the 3Q2J-ANF2OHCC complex. Significantly, the carboxyl group of this ligand allows the formation of a salt bridge with LYS44 and a hydrogen bond with ASP208 (Figure 6A,B). These interactions could compensate for the energetic penalty resulting from the desolvation of the polar carboxyl group introduced into the ligand.

### 2.7. Physicochemical, Pharmacokinetic, and Toxicological Predictions of ANF2OHC and ANF2OHCC

At this point, it was decided to compare some physicochemical parameters of ANF2OHC and ANF2OHCC with those of ANF and two antibiotics capable of reaching cytoplasmic bacterial targets: ciprofloxacin (CIP) and azithromycin (AZ), a fluoroquinolone and a macrolide, respectively. Supplementary Table S2 shows that the introduced polar groups allow a substantial increase in the topological polar surface area (TPSA) from 30.21 Å^2^ for ANF to 110.8 Å^2^ for both derivatives, which represents an intermediate value between TPSA values of CIP (180.08 Å^2^) and AZ (74.57 Å^2^). The LogP of ANF2OHC (2.843) and also that of ANF2OHCC (2.691) confirm their lower hydrophobicity compared to ANF (4.894), and intermediate between AZ (3.498) and CIP (−0.812). In addition, the LogD (LogP at physiological pH 7.4) of both derivatives also ranks between the values of the two antibiotics. Finally, the LogS which reflects the hydrosolubility of the molecules shows very close values of ANF2OHC (−3.86) and ANF2OHCC (−3.82) to that of AZ (−3.854). 

It was then evaluated whether the presence of polar groups in ANF2OHC and ANF2OHCC improves their pharmacokinetic profiles compared to ANF, Table 2. The analyses performed with SwissADME show that, compared to ANF; ANF2OHC and ANF2OHCC have a lower probability of crossing the BBB, which decreases the potential for adverse effects on the central nervous system. On the other hand, while ANF could be an inhibitor of the cytochromes CYP1A2 and CYP2C19, ANF2OHC would inhibit also CYP1A2 and CYP2D6, whereas ANF2OHCC has no potential to inhibit any class of cytochromes. In addition, considering that aminoglycosides, such as amikacin are excreted almost without undergoing metabolism [41] and do not inhibit cytochromes [42], there would be a low probability of interaction in case of simultaneous administration of this antibiotic and these derivatives through cytochrome-related interactions. 

Predictions performed with the Pro-Tox II server also reveal a better toxicological profile of ANF2OHC and ANF2OHCC compared to ANF considering their lower mutagenic and cytotoxic potential (Table 2). 

### 2.8. Target Fishing and Synthetic Accessibility Analyses

To complement the information previously obtained, target fishing analyses were carried out to identify potential human targets for ANF, ANF2OHC, and ANF2OHCC (Supplementary Tables S3–5, respectively). Analyses performed with Swiss Target Prediction show that compared to ANF2OHC and ANF2OHCC, ANF has higher likely to interact with human targets. These include the TYR and SER/ THR protein kinases, which is a reasonable prediction due to their high homology with APH3′-IIIa enzymes [12,43]. In particular, ANF has a 100% probability of interacting with human DNA-dependent protein kinase, whereas ANF2OHC and ANF2OHCC show a probability of 10 and 11%, respectively. Based on these predictions, there would be a lower risk of undesirable reactions caused by off-target effects of ANF2OHC and ANF2OHCC in this type of human enzymes.

Finally, the synthetic accessibility evaluated with SwissADME shows a value of 3.08 for ANF2OHC and 3.16 for ANF2OHCC on a scale of 1 (very easy) to 10 (very difficult), indicating that these compounds can be synthesized.

### 2.9. Molecular Dynamic Simulation Analyses

**Figure 7:**
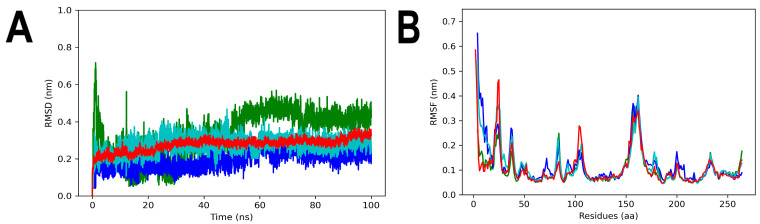
RMSD and RMSF analyses of apo-3Q2J and 3Q2J complexed with ANF, ANF2OHC and ANF2OHCC. (**A**) RMSD values. (**B**) RMSF values. The colors red, green, blue and cyan represented, respectively, apo-3Q2J, 3Q2J-ANF, 3Q2J-ANF2OHC, and 3Q2J-ANF2OHCC.

To examine the stability, interactions and conformational changes in the complexes formed by 3Q2J and selected flavones, molecular dynamics (MD) simulations were conducted. The stability of apo-3Q2J was initially assessed by calculating its RMSD, which remained consistently stable throughout the simulation time with an average value of 0.25 nm. However, among the complexes, 3Q2J-ANF showed stronger fluctuations and only stabilized in the final 45 ns of the simulation with an average RMSD value of 0.42 nm. On the other hand, the 3Q2J-ANF2OHCC and 3Q2J-ANF2OHC complexes have an average RMSD value of 0.28 nm and 0.21 nm, respectively until the end of the run (Figure 7A). 

The fluctuation analysis of amino acid residues revealed that apo-3Q2J and its complexes had higher flexibility in the regions between residues 23–29 (maximum RMSF value of 0.46 nm) and 157–162 (maximum RMSF value of 0.38 nm), which are located close to the nucleotide- and aminoglycoside-binding sites, respectively [12]. However, the presence of three ligands ANF, ANF2OHCC and ANF2OHC reduced the fluctuations in the region between residues 23–29 of 3Q2J (maximum RMSF of 0.27, 0.36 and 0.28 nm, respectively). Moreover, since 3Q2J lacks a substrate in its aminoglycoside binding site, the fluctuations in regions 157–162 were similar in all systems (Figure 7B). 

**Figure 8:**
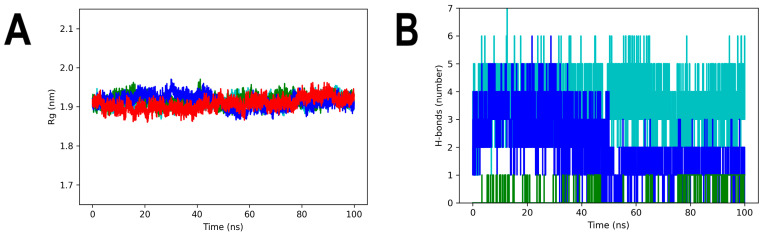
Rg and H-bonds analyses of 3Q2J complexed with ANF, ANF2OHC, and ANF2OHCC. (**A**) Rg values. (**B**) H-bonds number. The colors red, green, blue and cyan represented, respectively, apo-3Q2J, 3Q2J-ANF, 3Q2J-ANF2OHC, and 3Q2J-ANF2OHCC.

To investigate the compactness of each system over time, Rg analyses were conducted. When analyzing apo-3Q2J, it was observed that it had higher compactness than the three complexes at the beginning of the simulation. However, in the last 50 ns of the run, the four profiles became very similar, with an average Rg of 1.91 nm (Figure 8A). Notably, the 3Q2J-ANF2OHC complex exhibited a slightly higher Rg value (1.95 nm) during the first 50 ns compared to the other systems. This profile agrees with the changes observed in the RMSD values at the same time previously.

Regarding the H-bonds in the complexes, ANF established at most one interaction with 3Q2J and it occurred intermittently during the run time, which is to be expected due to the absence of polar substituents in its backbone. On the other hand, in the 3Q2J-ANF2OHC complex, a maximum of six H-bonds were detected. Five of them were maintained intermittently from 3 to 37 ns. However, in the last 50 ns of the run only two H-bonds were maintained (Figure 8B). Interestingly, this significant decrease in the number of H-bonds, again in the last 50 ns of the run, coincided with the changes observed in the RMSD and Rg analyses. Regarding the complex 3Q2J-ANF2OHCC, it can be observed that the H-bonds remained almost constant throughout the MD simulation with an average of four to five bonds and reaching a maximum of seven.

To examine more closely the interactions of ANF2OHC and ANF2OHCC with NBS of the 3Q2J, snapshots were taken at representative stages of the MD simulations. The analysis of the 3Q2J-ANF2OHC complex at 25 ns, Figure S4A, shows that at this point the ligand is more exposed to the solvent than at 100 ns, Figure S4B, which could explain the lower compactness of the complex at the beginning of the run. In contrast, in the interaction between 3Q2J- ANF2OHCC complex no drastic changes are observed at these same times, respectively, represented on Figure S4C,D. As the 3Q2J-ANF2OHC MD simulations progresses, the 2D interaction diagrams at 25, 50, 75, and 100 ns show that H-bonds contribute proportionally less to stabilize the complex compared to other types of interactions (Figure S5A–D). 

**Figure 9:**
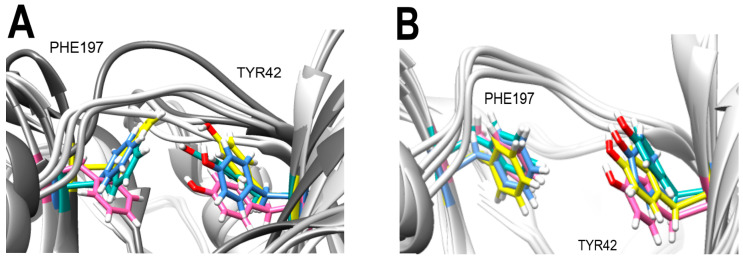
Overlap of the four molecular dynamics snapshots at different run times of 3Q2J complexed with (**A**) ANF2OHC and (**B**) ANF2OHCC. Amino acids TYR42 and PHE197 are represented in pink at 25 ns, green at 50 ns, blue at 75 ns and yellow at 100 ns.

Notably, during the first 25 ns of MD simulation of 3Q2J-ANF2OHC, PHE197 forms Pi–Pi stacking interactions with the C ring of the ligand (Figure S5A). However, at 50 ns, PHE197 establishes Pi–Pi T-shaped interactions with this same ring and with the chromone ring, and this interaction persists at 75–100 ns (Figure S5B–D). In fact, this change is accompanied by an approximately 90-degree turn that the phenyl ring of PHE197 undergoes from its conformation at 25 ns to that observed at 50, 75, and 100 ns (Figure 9A). On the other hand, the critical residue TYR42 undergoes fewer fluctuations. Although this amino acid does not interact with the ligand as represented at 25 ns snapshot, it is evident in the next three snapshots that it plays an essential role in the interaction with the benzo[h]chromen-4-one system. 

Concerning the 3Q2J-ANF2OHCC complex (Figure 9B), both PHE197 and TYR42 show less conformational changes, as evidenced by their permanent contribution in interactions with the phenyl ring and the benzo[h]chromen-4-one system, respectively. Additionally, these snapshots also suggest that all polar groups establish interactions throughout the all-time of the MD simulation (Figure S6A–D).

### 2.10. Binding Free Energy Calculation

**Figure 10:**
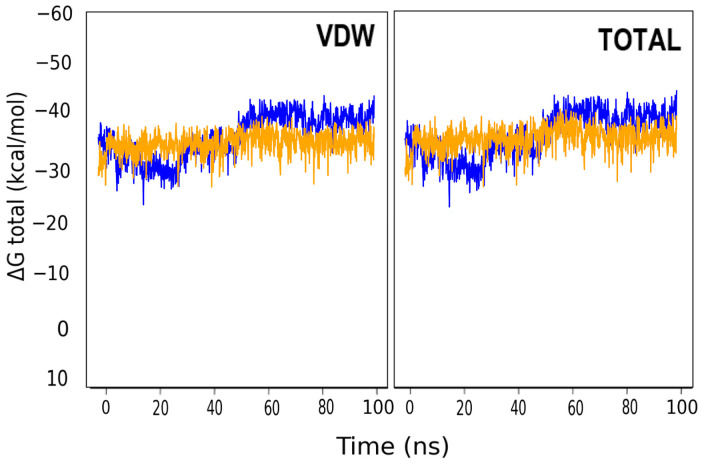
Binding free energy calculation of all run time of the MD simulation (100 ns) of 3Q2J-ANF2OHC and 3Q2J-ANF2OHCC complexes. The colors blue and orange represented, respectively, 3Q2J-ANF2OHC and 3Q2J-ANF2OHCC complexes. The calculations were conducted using gmx-MMPBSA-1.5.7 by analyzing 5000 snapshots.

To corroborate the results previously obtained, the free binding energy of the complexes formed by the enzyme with ANF, ANF2OHC, and ANF2OHCC were calculated. The results presented in Table 3 demonstrate that the complex with ANF2OHC and ANF2OHCC have higher total free energy compared to that of ANF. The decomposition of the binding free energy shows that in the complexes the largest contribution comes from ΔE (Vdw) and to a lesser extent from ΔE (Ele) and ΔE (NPolar), while there is a larger unfavorable effect of ΔE (PB) for the complex with ANF2OHC compared to that formed with ANF2OHCC and also ANF. In addition, throughout the full analysis time, the latter complex showed smaller variations in binding energy compared to that formed by ANF2OHC, Figure 10.

Although the ΔG (Total) of 3Q2J-ANF2OHC at the beginning of the analysis was less negative, at no time did it decrease to zero kcal/mol, which confirms that the ligand remains bound to the enzyme throughout the analysis time. On the other hand, it is observed that the ΔG (Total) of the 3Q2J-ANF2OHCC complex maintains a constant profile from all the time analyzed. Collectively, these results demonstrate that interactions due to the alpha-naphthoflavone backbone play a major role in the binding energy of both derivatives with EfAPH3’-IIIa.

## 3. Methods

### 3.1. Target and Ligands Preparation 

For the present study, the 3D X-ray diffraction structure of the enzyme EfAPH3’-IIIa was selected from the RCSB Protein Data Bank (PDB ID: 3Q2J co-crystalized with CKI-7 [12]) and downloaded in March 2022. The 3Q2J crystal was prepared using the Dock Prep module of UCSF Chimera-1.16 [44] using default parameters, then converted into PDBQT format using the AutoDockTools-1.5.6 [45].

The chemical structures of the 462 flavones were retrieved from the ChemSpace database of small molecules and downloaded in SDF format using the software DataWarrior-5.5.0 [46] in March 2022. Subsequently, the hydrogens were assigned to the flavones at pH 7.4 followed by minimization steps of these structures with the MMFF94 force field using the conjugate gradient algorithm with default parameters and transformed into PDBQT using the Open Babel-3.1.1 software [47]. Molecules designed from the most promising ligand were generated with the Marvin JS web server and subsequently prepared for molecular docking following the same protocol mentioned above.

### 3.2. Molecular Docking-Based Virtual Screening 

The virtual screening analysis was based on the molecular docking approach using the AutoDock Vina-1.1.2 software [48] with an exhaustiveness of eight, the other parameters were kept according to the software recommendations. These analyses were performed considering the co-crystalized ligand coordinates in the NBS of the enzyme: 27.9, 9.6 and 70.7 (x, y, and z axes, respectively [12]) and grid dimensions of 30 × 30 × 30 Å. In the blind docking analysis, the same coordinates were used but the grid dimensions were 50 × 50 × 50 Å. Before starting the virtual screening, the ability of the AutoDock Vina to reproduce the crystallographic pose of CKI-7 in the NBS (nucleotide-binding site) in 3Q2J was validated. The DockRMSD web server [49] was used to calculate the RMSD values.

### 3.3. Physicochemical, Pharmacokinetic, Toxicological and Target Fishing Analyses 

The analyses of the physicochemical properties of the most promising ligands were performed with the ADMET lab-2.0 server [50]. The pharmacokinetics and synthetic accessibility predictions were carried out using the Swiss-ADME server [51]. The hepatotoxicity, carcinogenic, immunotoxicity, mutagenicity, and cytotoxicity were analyzed by using the ProTox-II server [52]. Target fishing analyses for the selected ligands were performed with the Swiss Target Prediction server [53]. 

### 3.4. Molecular Dynamic Simulations 

MD simulation analyses were performed to deepen the understanding of the interactions and to evaluate the stability of the 3Q2J complexed with the selected ligands. All analyses were performed using GROMACS-2021.1 software [54] and using all-atom CHARMM 36 force field [55]. The first step carried out was the solvation of the system using transferable intermolecular potential water model 3P (TIP3P), selecting a periodically corrected cubic box using a minimum distance of 1 nm. Subsequently, the system was neutralized by the addition of Na^+^ and Cl^−^ ions, and 100,000 energy minimization steps were achieved using the steepest descent algorithm to eliminate initial steric shocks. The system was equilibrated for 500 and 2500 ps at 310 K and 1 bar pressure in the NVT and NPT matrices, respectively, and the productions were conducted during 100 ns long and coordinates were saved every 10 ps. All procedures were conducted using the Leap-frog algorithm and Berendsen coupling to control pressure and temperature [56]. The Particle Mesh Ewald (PME) algorithm was used to analyze the long-range electrostatic interactions [57] and LINCS algorithm implementation was used to regulate the covalent bonds [58].

### 3.5. Binding Free Energy Calculation

To complement the MD simulation analyses, free energy calculations were performed with the complexes formed between 3Q2J and the selected ligands using the MMPBSA method [59] based on a single trajectory analysis with gmx-MMPBSA 1.5.7 software [60]. To perform the analyses, information was extracted from representative times of the execution of each MD simulation. The parameters that were used to calculate the free energies were inp = 1, istrng = 0.15, and indi = 2, the other parameters were kept according to the software recommendations.

### 3.6. Visualizations of Molecular Docking and Molecular Dynamics Results 

The 2D diagrams and 3D representations were obtained using the software Discovery Studio Visualizer-2021 and UCSF Chimera, respectively. MD simulation results were visualized with GROMACS scripts in conjunction with Python scripts using the NumPy, Pandas, Matplotlib, Seaborn, and Pytraj libraries. The RMSD results were generated from the alpha-carbon of the protein in the presence or absence of the ligands. RMSF, Rg, and H-bonds representations were generated from the protein in the presence or absence of the ligands.

## 4. Conclusions

Due to the limited efficiency of existing antibiotics to circumvent *E. faecalis* resistance events, especially in hospital environments, there is an urgent need to find molecules that act as adjuvants to traditional antibiotics. In this in silico approach, the ability of 462 flavones to interact in the nucleotide-binding site (NBS) of EfAPH3’-IIIa was tested. The main results show that 2-phenyl-4H-benzo[h]chromen-4-one, ANF, an alpha-naphthoflavone, showed the best score over the whole flavone dataset. The polar groups added on the backbone of ANF resulted in the derivatives, ANF2OHC and ANF2OHCC, with higher binding free energy, improved stability in MD simulation analyses, and better pharmacokinetic and toxicological profiles. In addition, the alpha-naphthoflavone backbone participates in interactions that are largely responsible for its high affinity for the active site of the enzyme, including interactions with key amino acids for its activity. Taken together, these results present new potentials adjuvants to aminoglycosides and reveal that the alpha-naphthoflavone core may be a promising new scaffold for the development of APH3’-IIIa inhibitors of *E. faecalis* to restore its sensitivity to aminoglycosides.

## Figures and Tables

**Table 1:** Structure and binding energies of the best-scored flavones. BS-1 (CSSB00000067355), BS-2 (CSSB00000067363), BS-3 (CSSB00160825754), BS-4 (CSSB00011876001), BS-5 (CSSB00102592853) and CKI-7. The analyses were conducted with AutoDock Vina-1.1.2.

Score	Structure	Binding Energy (kcal/mol)
BS-1	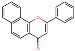	−10.5
BS-2	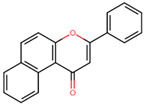	−10.4
BS-3	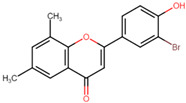	−10.4
BS-4	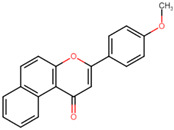	−10.3
BS-5	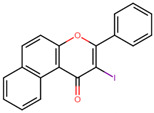	−10.3
CKI-7	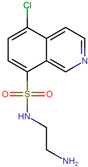	−8.2

**Table 2:** Comparative pharmacokinetics and toxicological analyses of ANF, ANF2OHC and ANF2OHCC. Pharmacokinetics analyses were performed with the SwissADME web server while toxicological analyses were performed with the Pro-Tox II web server. Values in parentheses refer to probability.

**Pharmacokinetic Analyses**			
	**ANF**	**ANF2OHC**	**ANF2OHCC**
GI absorption	High	High	High
BBB permeant	Yes	No	No
P-gp substrate	No	No	No
CYP1A2 inhibitor	Yes	Yes	No
CYP2C19 inhibitor	Yes	No	No
CYP2C9 inhibitor	No	No	No
CYP2D6 inhibitor	No	Yes	No
CYP3A4 inhibitor	No	No	No
**Toxicological analyses**			
	**ANF**	**ANF2OHC**	**ANF2OHCC**
Hepatotoxicity	Inactive (0.70)	Inactive (0.72)	Inactive (0.70)
Carcinogenicity	Active (0.69)	Inactive (0.58)	Inactive (0.63)
Immunotoxicity	Inactive (0.85)	Inactive (0.81)	Inactive (0.75)
Mutagenicity	Inactive (0.54)	Inactive (0.74)	Inactive (0.71)
Cytotoxicity	Active (0.75)	Inactive (0.81)	Inactive (0.74)

**Table 3:** Binding free energy calculation of ligands extracted from the single trajectory of MD simulation analyses of the 3Q2J-ANF, 3Q2J-ANF2OHC, and 3Q2J-ANF2OHCC complexes. The calculations were conducted on the final 40 ns of the run using gmx-MMPBSA-1.5.7 by analyzing 4000 snapshots.

Energy Decomposition (Kcal/mol) ± SD			
	ANF	ANF2OHC	ANF2OHCC
ΔE (Vdw)	−29.7 ± 3.8	−42.8 ± 3.1	−35.0 ± 3.2
ΔE (Ele)	−1.0 ± 0.8	−3.9 ± 1.0	−5.7 ± 1.2
ΔE (PB)	2.9 ± 0.7	6.6 ± 0.8	6.2 ± 0.8
ΔE (NPolar)	−3.0 ± 0.2	−4.1 ± 0.1	−4.0 ± 0.1
ΔG (Total)	−29.3 ± 3.9	−44.2 ± 2.9	−38.4 ± 3.0

## Data Availability

All data generated or analyzed during this study are included in this published article and its supplementary information files.

## Supplementary Materials

The following supporting information can be downloaded at: https://www.mdpi.com/article/10.3390/microorganisms11092351/s1, Figure S1: Re-docking of CKI-7 and Hydrogen bond molecular surface representation of ANF on nucleotide binding site of 3Q2J; Figure S2: Superposition of the site-directed and blind docking results of ANF2OHC and ANF2OHCC on the NBS of 3Q2J; Figure S3: Superposition of docked poses on the NBS of 3Q2J; Figure S4: Snapshots of the ANF2OHC and ANF2OHCC highlight the pose in the NBS of the 3Q2J during molecular dynamics simulations; Figure S5: Analysis of 2D diagrams of 3Q2J- ANF2OHC complex interactions over 100 ns of molecular dynamics simulations; Figure S6: Analysis of 2D diagrams of 3Q2J- ANF2OHCC complex interactions over 100 ns of molecular dynamics simulations, Table S1: Molecular docking-based virtual screening of 462 flavones on the nucleotide-binding site of EfAPH(3’)-IIIa; Table S2: Physicochemical properties of ANF, ANF2OHC and ANF2OHCC compared to AZ and CIP predicted using ADMET lab-2.0 server; Table S3: ANF target prediction using Swiss Target Prediction; Table S4: ANF2OHC target prediction using Swiss Target Prediction; Table S5: ANF2OHC target prediction using Swiss Target Prediction.
