# Supplementary material for: Alpha-Naphthoflavone as a Novel Scaffold for the Design of Potential Inhibitors of the APH(3’)-IIIa Nucleotide-Binding Site of Enterococcus faecalis
Source: Microorganisms. 2023 Sep 20;11(9):2351. doi: 10.3390/microorganisms11092351 (PMC10535617; doi:10.3390/microorganisms11092351)
Supplement: Supplementary file 1 [file microorganisms-11-02351-s001.zip › microorganisms-2494506-supplementary.pdf]

Supplementary material

Alpha-naphthoflavone as a novel scaffold for the design of potential inhibitors of the APH(3')-IIIa nucleotide-binding site of *Enterococcus faecalis*

Juliana Carolina Amorim, Juan Marcelo Carpio*

Academic Unit of Health and Wellness, Catholic University of Cuenca, Azuay, Ecuador

Corresponding author: juanbiomarce@gmail.com

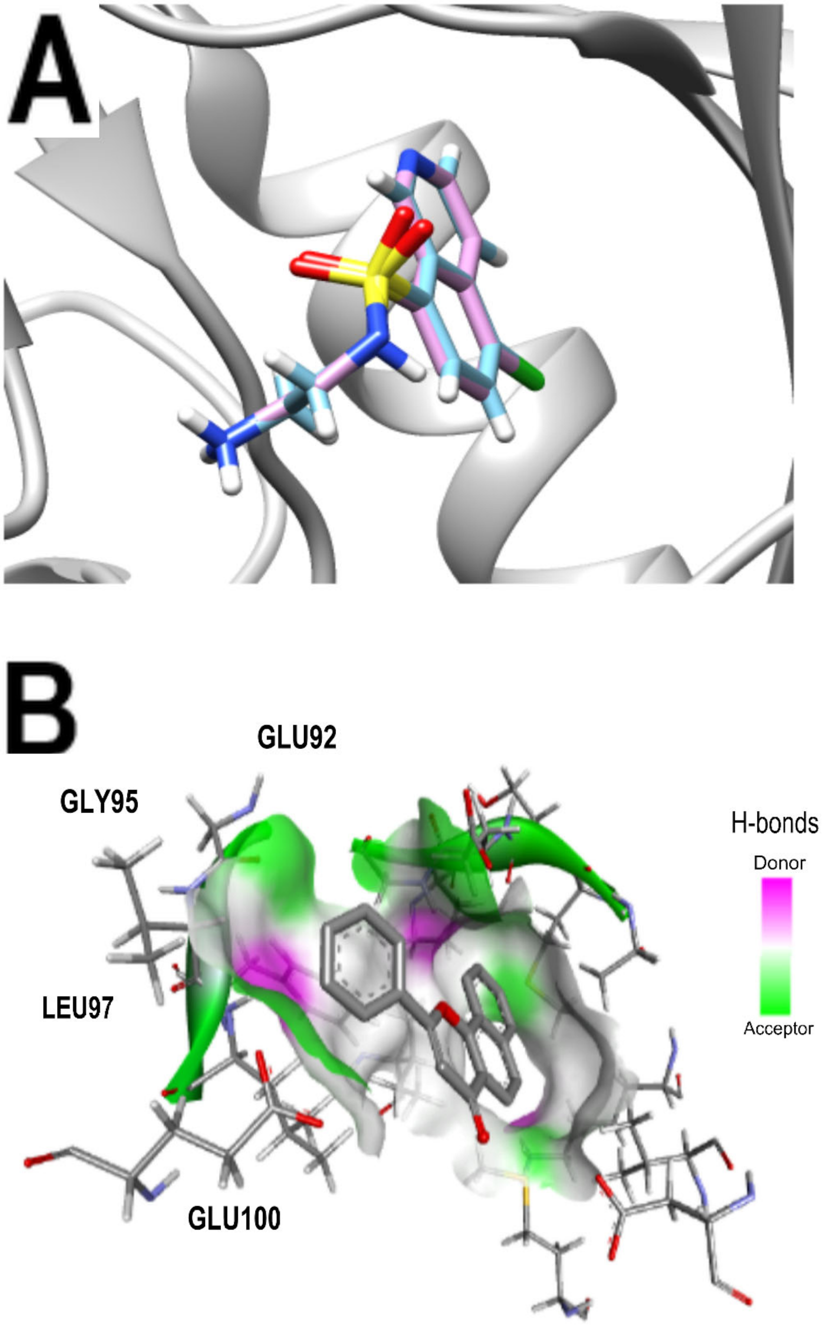

Fig. S1. Re-docking of CKI-7 and Hydrogen bond molecular surface representation of ANF on the nucleotide-binding site of 3Q2J. A) Ribbon representation of CKI-7 redocked on nucleotide binding site of 3Q2J and B) Hydrogen bond molecular surface representation of nucleotide binding site of 3Q2J complexed with ANF.

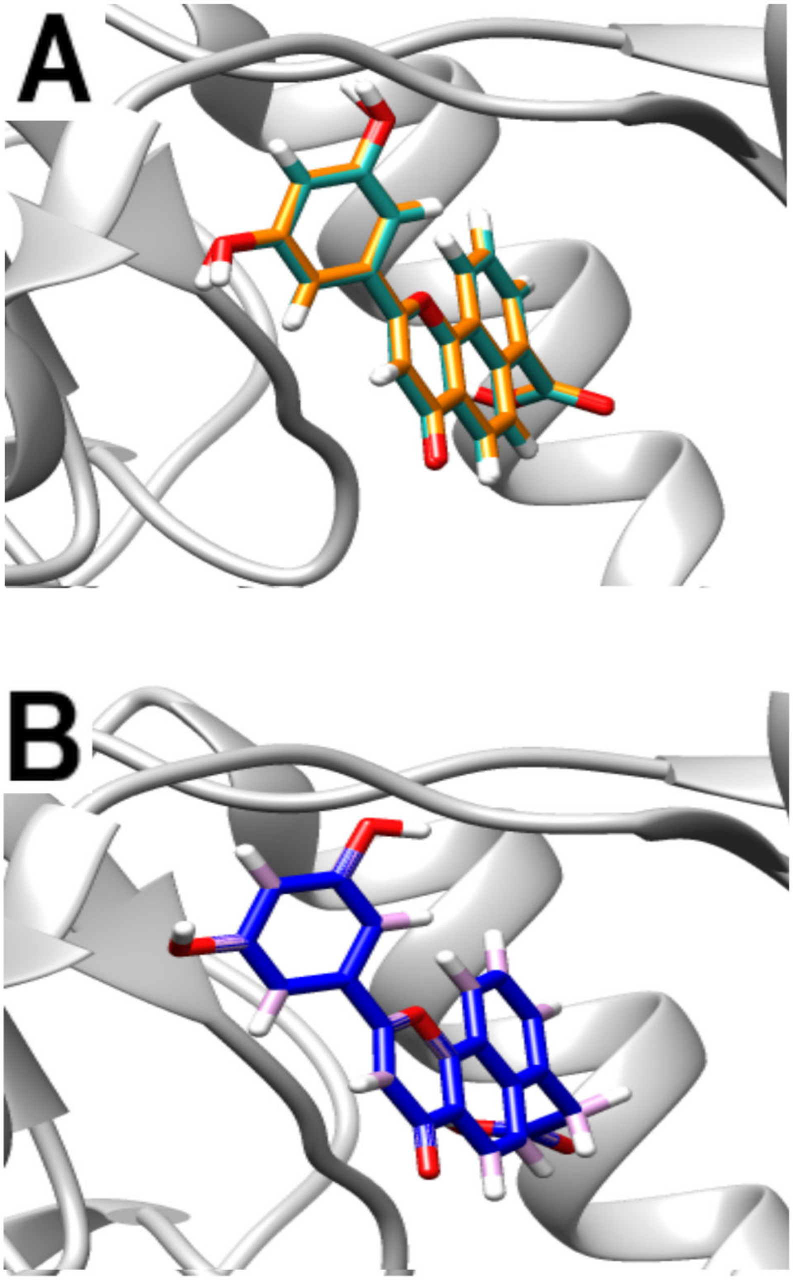

Fig. S2. Superposition of the site-directed and blind docking results of ANF2OHC and ANF2OHCC on the NBS of 3Q2J. A) Ribbon representation of 3Q2J with the site-directed and blind docking poses of ANF2OHC. B) Ribbon representation of 3Q2J with the site-directed and blind docking poses of ANF2OHCC. In orange is depicted ANF2OHC docked in a box dimension of 30 x 30 x 30 Å, and in light green, docked in a box of 50 x 50 x 50 Å. In blue is depicted ANF2OHCC docked in a box dimension of 30 x 30 x 30 Å, and in light pink, docked in a box of 50 x 50 x 50 Å.

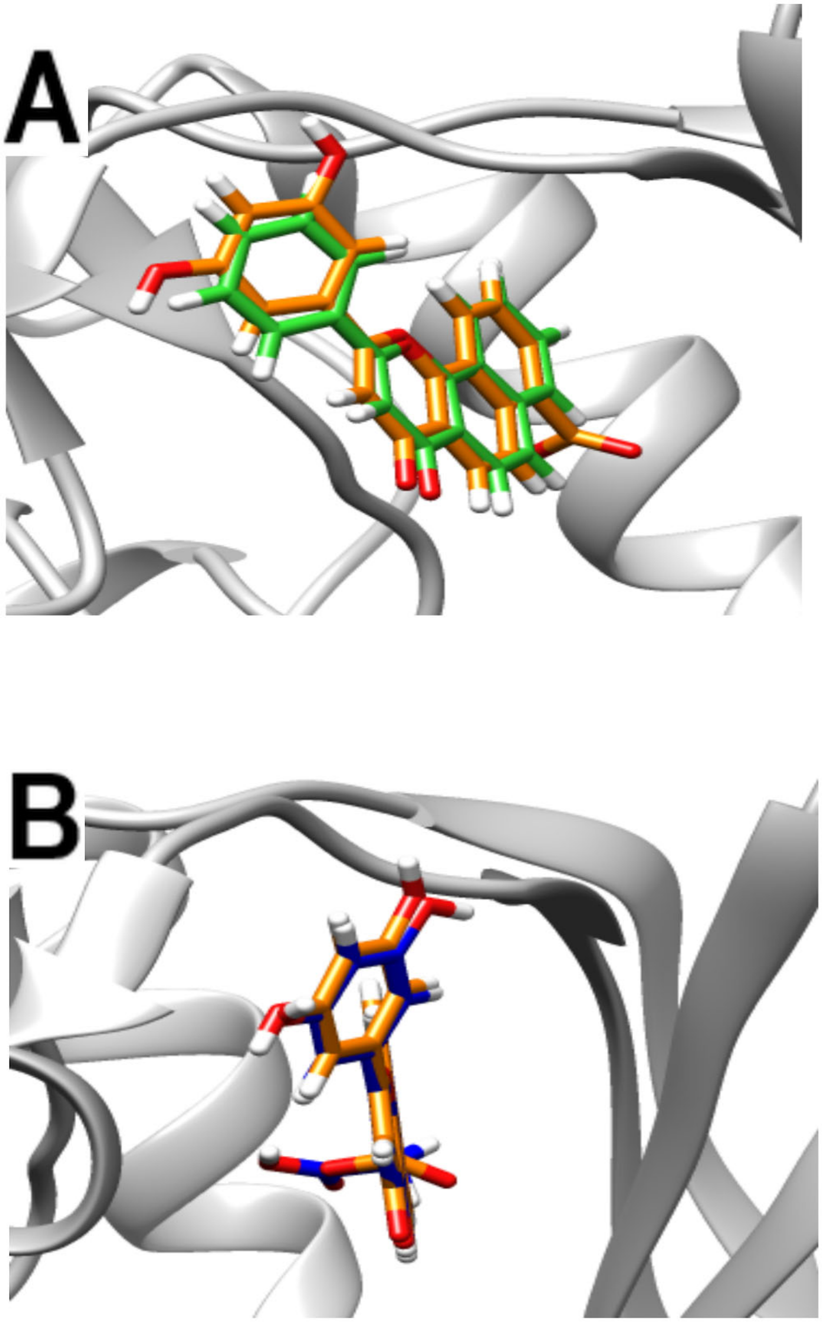

Fig. S3. Superposition of docked poses on the NBS of 3Q2J. A) Ribbon representation of superposition of ANF and ANF2OHC on the nucleotide-binding site of 3Q2J. B) Ribbon representation of superposition of NF2OHC and ANF2OHCC on the nucleotide-binding site of 3Q2J.

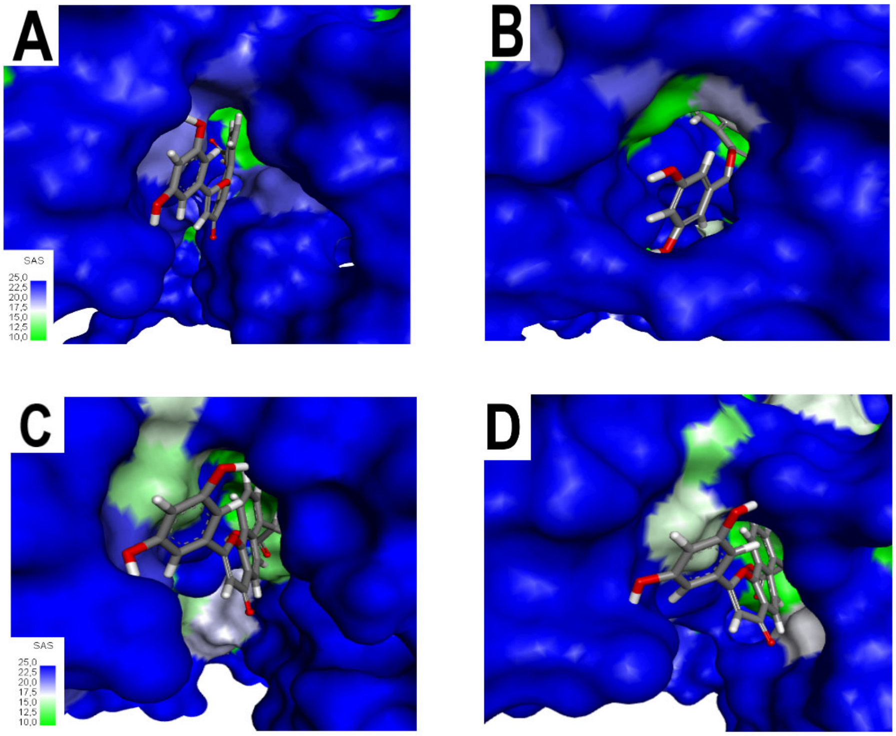

Fig S4. Snapshots of the ANF2OHC and ANF2OHCC highlight the pose in the NBS of the 3Q2J during molecular dynamics simulations. A) Snapshot of 25 ns of 3Q2J-ANF2OHC MDS and B) 100 ns. C) Snapshot of 25 ns of 3Q2J-ANF2OHCC MDS and D) 100 ns.

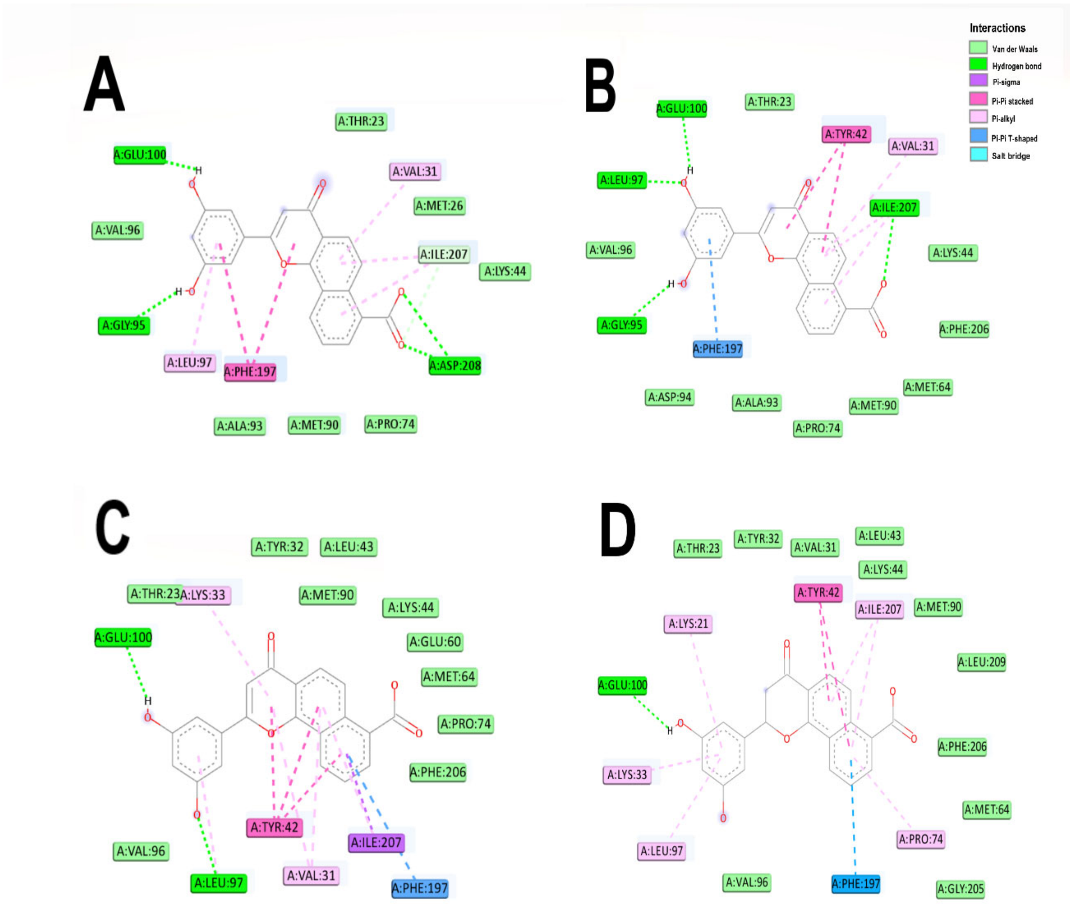

Fig. S5. Analysis of 2D diagrams of 3Q2J- ANF2OHC complex interactions over 100 ns of molecular dynamics simulations. A) Snapshot of 25 ns, B) 50 ns, C) 75ns and D) 100 ns.

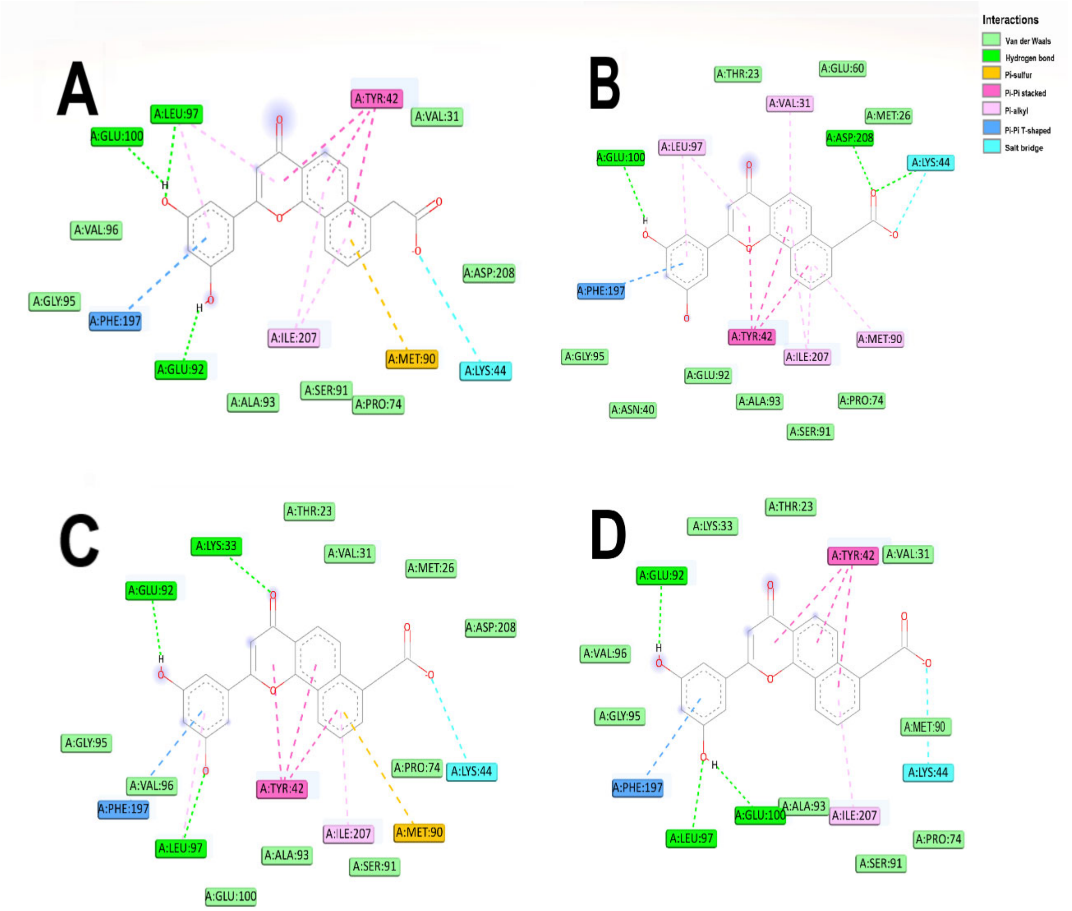

Fig. S6. Analysis of 2D diagrams of 3Q2J- ANF2OHCC complex interactions over 100 ns of molecular dynamics simulations. A) Snapshot of 25 ns, B) 50 ns, C) 75ns and D) 100 ns.
